# Supplementary figures and images for: Evaluating the appropriate oral lipid tolerance test model for investigating plasma triglyceride elevation in mice
Source: PLoS One. 2020 Oct 6;15(10):e0235875. doi: 10.1371/journal.pone.0235875 (PMC7537863; doi:10.1371/journal.pone.0235875)

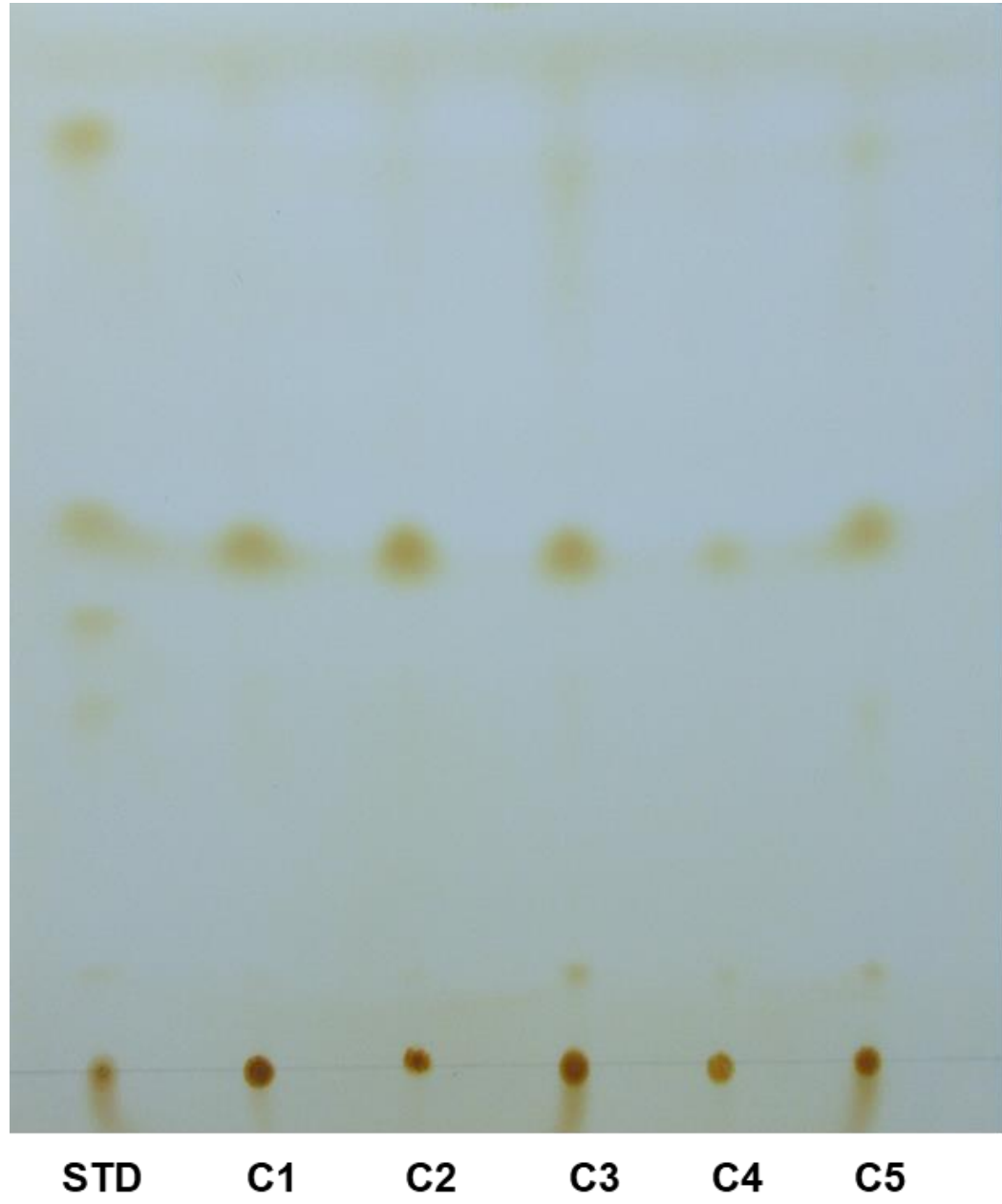

**Control**

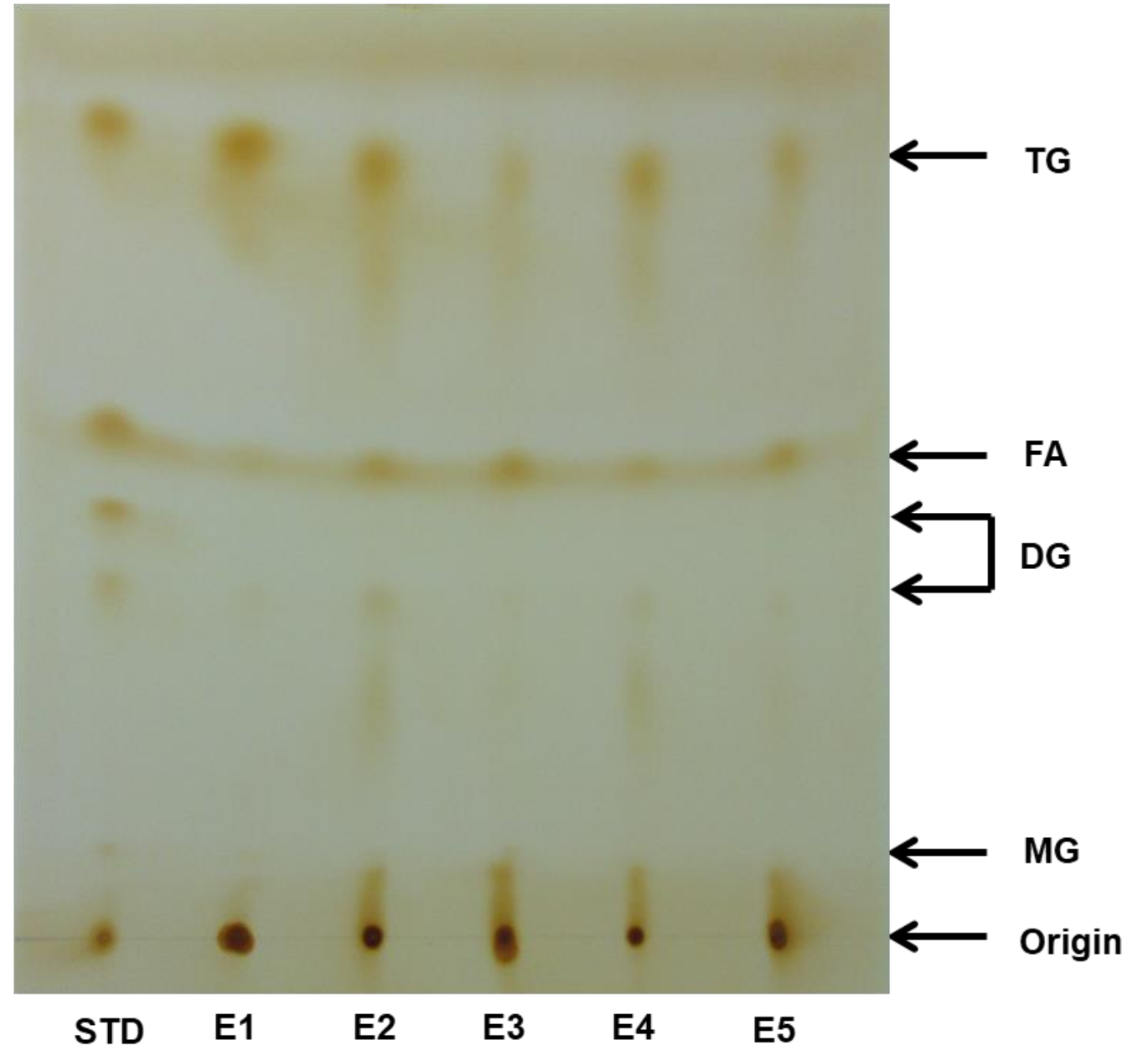

**EGCG**

Supplement: S1 Fig — The S1 Fig supports Fig 7. The S1 Fig shows the effect of EGCG on intestinal lipids source following the lipids administration in ddY mice. Extracted lipids in the small intestine were separated on a HPTLC. STD, a mixture of standard lipids, contained a mixture of reagent-grade triolein (TG), diolein (DG), monoolein (MG), and oleic acid (FA). The spot was developed on a plate with hexane/diethyl ether/acetic acid (60:40:1, v/v). The developed spots of each lipid were visualized using iodine. (PDF) [file pone.0235875.s001.pdf]
